# Feeding Patterns and Xenomonitoring of Trypanosomes among Tsetse Flies around the Gashaka-Gumti National Park in Nigeria

**DOI:** 10.1155/2016/1591037

**Published:** 2016-02-15

**Authors:** Solomon Ngutor Karshima, Idris A. Lawal, Oluseyi Oluyinka Okubanjo

**Affiliations:** ^1^Department of Animal Health, Federal College of Animal Health and Production Technology, PMB 001, Vom, Nigeria; ^2^Department of Veterinary Parasitology and Entomology, Ahmadu Bello University, PMB 1045, Zaria, Nigeria

## Abstract

In order to understand the epidemiology of trypanosomoses in Gashaka-Gumti National Park, Nigeria, we determined the density, infection rates, and feeding patterns of tsetse flies using biconical traps, ITS, and mitochondrial cytochrome b PCRs. A total of 631 tsetse flies were captured, of which 531 (84.2%) and 100 (15.8%) were analyzed for trypanosomes and blood meals, respectively. Tsetse distribution varied significantly (*p* < 0.05) across study sites with average trap and daily catches of 4.39 and 26.34, respectively. Overall tsetse infection rate was 5.08% and ranged between 3.03% and 6.84% across study sites. We identified 10* T. brucei*, 3* T. congolense savannah, *2* T. congolense forest*, and 2 mixed infections among the 13 pools made from the 27 flies positive for trypanosomes with light microscopy. The distribution of vertebrate blood meals in tsetse flies varied significantly (*p* < 0.05) and ranged between 6.0% and 45% across hosts. We also observed dual feeding patterns involving at least 2 hosts in 24% and multiple feeding involving at least 3 hosts in 17% of the flies. We observed predominance of* G. palpalis *which also recorded higher infection rate.* T. brucei *was more prevalent among tsetse flies. Tsetse flies fed predominantly on cattle and less frequently on humans and also showed mixed feeding habits.

## 1. Introduction

Tsetse flies are large biting and blood-feeding flies of great economic, veterinary, and medical importance, due to their ability to transmit African animal and human trypanosomoses. Tsetse-transmitted trypanosomoses occur in 38 sub-Saharan African countries with averages of 15,000 human cases and one million cattle deaths reported yearly, exposing over 70 million people and 160 million cattle to the risk of infection in the region [1]. These vectors are distributed over wide range of habitats covering about 10 million square kilometers of potential grazing and farming lands in sub-Saharan Africa [2].

Tsetse abundance and feeding behaviours determine the degree of vector-host contact and may have a serious impact on the risk of pathogen transmission. The degree of contacts between vectors and vertebrate hosts is an important determinant of their vectoral capacity and is determined by the vector feeding patterns on its hosts. As vectors of human and animal trypanosomoses, the epidemiology of these diseases is determined largely by their abundance, density, and feeding behaviours [3].

The importance of vector feeding patterns in the epidemiology of diseases has been recognized for quite a long time. However, majority of the traditional approaches used earlier were serologically based and included haemagglutination crystallization, agglutination reactions, passive haemagglutination test, immunohistochemical methods [4, 5], precipitin test [6, 7], and enzyme linked immunosorbent assay [8–10]. Despite the level of successes achieved by these traditional approaches, limitations ranging between high percentage of false positives, low sensitivity, species cross infectivity, the need for producing specific antibodies to several species, and the inability to discover unpredicted hosts were reported [11].

The development of molecular based techniques for the identification of vertebrate host blood meals provides more convenient approaches and has already been employed in the detection of host blood meals in several arthropod vectors including tsetse flies [12], ticks [13, 14], triatomine bugs [15], and mosquitoes [16, 17]. In order to understand the epidemiology of trypanosomosis in the Gashaka-Gumti National Park in Nigeria, we trapped and studied the feeding patterns of tsetse flies using the mitochondrial cytochrome b PCR and xenomonitored trypanosomes in tsetse flies using ITS PCR.

## 2. Materials and Methods

### 2.1. Study Area

The study was carried out in Gashaka-Gumti National Park which is located in Taraba State, Northeastern Nigeria, between latitudes 6°55′ and 6°17′N and longitudes 11°13′ and 11°21′E. It is the largest national park in Nigeria, covering 6402 km^2^ and consisting of both savannah grassland and montane forest vegetation. Altitude ranges from 457 meters (1,499 ft) to 2419 meters (7,936 ft). It is an important water catchment area for the Benue River with abundant river flow even during the markedly dry season. Enclaves for local Fulani pastoralists exist within the park's boundary allowing for farming and grazing. The reserve also contains a wide range of wild fauna of over 103 different species [18]. The major occupation of the inhabitant is agriculture.

### 2.2. Study Design

We conducted a cross-sectional study in four randomly selected villages (Burto, Gindin Dutse, Goje, and Serti) located at least 5–10 km apart around the Gashaka community taking cognisance of their relationship with the Gashaka-Gumti National Park which is a tourist centre. We identified forested and riverine areas where 6 traps were mounted 100 meters apart two times weekly for four months in each of the four villages.

### 2.3. Tsetse Trapping, Identification, and Dissection

Tsetse flies were trapped by the use of biconical traps as described by Charllier and Laveissiere [19]. Six traps were mounted 100 meters apart along streams twice every week between July and October, 2014. Traps were emptied every 24 hours and the flies were identified using morphological characteristics as described by Murray et al. [20]. Following identification, a proportion of the collected tsetse flies were dissected using dissection microscope to check for vector stages of trypanosomes on the field while the most engorged were selected for blood meal identification. Flies that were positive following dissection and the engorged were all preserved at −80°C until needed for PCR.

### 2.4. DNA Extractions

Blood meal DNA extraction was done using the modified salt procedure earlier described by Norris et al. [21]. Tsetse abdomen was detached from the thorax and head and homogenized in 100 *μ*L of extraction buffer containing 0.1 M NaCl, 0.2 M sucrose, 0.1 M Tris-HCl, 0.05 M EDTA, pH 9.1, and 0.5% sodium dodecyl sulfate (SDS) and incubated at 65°C for one hour. Extracted DNA was then stored at −20°C until needed for polymerase chain reaction.

Trypanosome DNA was extracted using GeneJET genomic DNA extraction kit (Thermo Scientific, Germany). Midguts of positive tsetse flies were pooled together in twos and homogenized and 200 *μ*L of the homogenate was lysed by adding 400 *μ*L of lyses solution and 20 *μ*L of proteinase K as recommended by the manufacturer. Extracted DNA was stored at −20°C until needed for PCR.

### 2.5. PCR Identification of Blood Meals

We conducted multiplex PCR with four forward primers and a universal reverse primer to identify mitochondrial cytochrome b of vertebrate hosts in blood meals of tsetse flies (Table 1). Cycling conditions were as follows: denaturation at 98°C for 10 seconds to activate the phusion flash II DNA polymerase, followed by 36 cycles at 98°C for 1 second, annealing for 50 seconds at temperatures of 58.5°C, 59.5°C, 62.0°C, and 62.5°C for cattle, dog, pig, and human primers, respectively. Extension was at 72°C for 40 seconds and final extension at 72°C for 5 minutes according to the manufacturers' instructions. All amplified products were analyzed by electrophoresis in a 2% agarose gel and UV illumination after ethidium bromide staining.

### 2.6. PCR Identification of Trypanosomes

ITS-1 PCR was used to identify the species of trypanosomes in the tsetse flies using primer sets with the sequences 5′-CCGGAAGTTCACCGATATTG-3′ (forward) and 5′-TTGCTGCGTTCTTCAACGAA-3′ (reverse) designed by [22]. PCR conditions were as follows: an initial denaturation step for 10 seconds at 98°C to activate the phusion flash II DNA polymerase, four cycles of amplification with 1-second denaturation at 98°C, 5-second hybridization at 58°C, and 15-second elongation steps at 72°C; eight cycles of amplification with 1-second denaturation at 98°C, 5-second hybridization at 56°C, and 15-second elongation steps at 72°C; 23 cycles of amplification with 1-second denaturation at 98°C, 5-second hybridization at 54°C, and 15-second elongation steps at 72°C; and a final extension step of 5 minutes at 72°C as described by the manufacturer.

### 2.7. Data Analysis

Data collated were analyzed using Graph-Pad Prism 4.0. Infection rates were calculated by dividing the number of infected tsetse flies by the total number of flies we dissected and expressed as percentages. Average tsetse catch per day was determined by summing the daily catch per week and dividing by the number of days traps were mounted for the week while average catch per trap was determined by summing daily catch and dividing by the number of traps. We employ the Chi square (*χ* ^2^) and Fishers exact test where appropriate to determine variations in infection rates and the distribution of* Trypanosoma* species. The Analysis of Variance (ANOVA) was also employed to determine variations in the distribution of mammalian blood in tsetse blood meals and values of *p* < 0.05 were considered significant.

## 3. Result

A total of 631 tsetse flies were captured with average trap and daily catches of 4.39 and 26.34, respectively. We dissected 531 (84.2%) of the flies to check for the vector stages of trypanosomes while the remaining 100 (15.8%) were analyzed for vertebrate sources of tsetse blood meals. Overall tsetse infection rate was 5.08% while those in relation to study sites were 6.84%, 4.65%, 3.03%, and 4.96% for Burto, Gindin Dutse (G/Dutse), Goje, and Serti, respectively (Table 2).

Of the 2 species of tsetse flies captured, 408 (64.7%) were* Glossina palpalis* while 223 (35.3%) were* G. tachinoides*. Tsetse infection rates of 5.5% (19/347) and 4.3% (8/184) were revealed by* G. palpalis* and* G. tachinoides,* respectively (Table 3). The 27 tsetse flies positive for trypanosomes using light microscopy were distributed into 12 pools of two flies each and a pool of three flies yielding 13 pools which were subjected to the ITS PCR. We identified* Trypanosoma brucei* in 10,* Trypanosoma congolense* savannah in 3, and* Trypanosoma congolense* forest in 2 of the pools. Two of the pools revealed mixed infections of* Trypanosoma brucei* and* Trypanosoma congolense* (Table 4).

**Figure 1:**
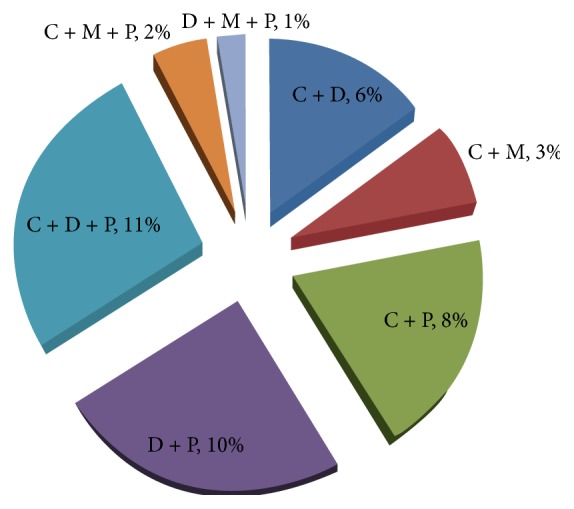
Distribution of mammalian blood in tsetse flies that fed on at least 2 hosts [C + D (cattle and dog mixed blood), C + M (cattle and man mixed blood), C + P (cattle and pig mixed blood), D + P (dog and pig mixed blood), C + D + P (cattle, dog, and pig mixed blood), C + M + P (cattle, man, and pig mixed blood), and D + M + P (dog, man, and pig mixed blood)].

Of the 100 engorged tsetse flies subjected to the mitochondrial cytochrome b PCR to identify sources of tsetse blood meals, 45, 17, 6, and 32 fed on cattle, dog, man, and pig, respectively (Table 5). In addition, 24% of the flies fed on at least 2 vertebrate hosts, 17% fed on at least 3 vertebrate hosts, and 1%, 5%, 22%, and 31% of the flies fed on man, dog, pig, and cattle, respectively (Table 5). The distribution of dual feeding among the 24 tsetse flies that fed on at least 2 vertebrate hosts was 15%, 7%, 20%, and 24% for flies that fed on cattle-dog, cattle-man, cattle-pig, and dog-pig, respectively. Of the 17 tsetse flies that fed on at least 3 vertebrate hosts, 27%, 5%, and 2% fed on cattle-dog-pig, cattle-man-pig, and dog-man-pig, respectively (Figure 1).

## 4. Discussion

The species of tsetse flies we reported in our study were earlier reported in the same region by Karshima et al. [23] and in other parts of Nigeria [24, 25] and were among the first eleven species of tsetse flies reported in Nigeria [26].* Glossina palpalis* was predominantly higher than* G. tachinoides* in line with earlier reports [25, 27, 28].* Glossina palpalis* usually has preference for thick forested areas with high temperatures and humidity for efficient breeding. Considering the site we studied which has similar kind of vegetation we presume that the vegetation may be a reason for the predominance of this species of tsetse fly.

We observed an overall average daily tsetse catch per trap of 4.39 which falls within the range of 0.61–8.1 reported by other workers [29–31]. We expected a higher average daily catch considering the game reserve terrain in which we conducted our study and reports that confirm tsetse abundance during the rainy season [31]. We attributed our size of catch to the consecutive rainfall we observed during the trapping as tsetse activities are grossly reduced during rain and cold weather.

The location of Gashaka-Gumti National Park in Serti and its close proximity to Burto may explain the higher tsetse catch we recorded in these two areas considering the fact that the park may serve as suitable habitat for tsetse flies and provide sources of blood meals for these flies. This may also explain the higher infection rates in these two areas especially with the expected increased tsetse animal contact with over 103 wildlife species and livestock grazing in the area [18]. The mixed infections of* T. brucei* and* T. congolense* observed may be due to tsetse acquiring the infection from animals carrying the mixed infection in nature, from two different animal sources during feeding or even from the pools we made in our study.

Tsetse flies that were positive for trypanosomes by the use of light microscopy were pooled together before being subjected to PCR to increase the quantity of DNA in the samples and thus increase the chances of PCR detection. The 2 species of trypanosomes identified in tsetse flies are not reported for the first time in this region, suggesting that they are endemic. Of the* Trypanosoma* species identified* T. brucei* was most prevalent among tsetse flies indicating that they may be the commonest species infective to the domestic animals and wildlife species in the region as earlier documented [23, 32, 33]. Majority of trypanosome infections were observed among* Glossina palpalis* contrary to earlier reports by Desta et al. [31]. This variation may not be unconnected with factors such as differences in tsetse feeding frequencies, feeding patterns, and vectoral capacity.

The feeding pattern of tsetse vector is an important instrument in understanding the epidemiology of human and animal trypanosomoses [3]. In our study, we detached engorged tsetse abdomens from the thorax and head before PCR to reduce contamination of blood meal DNA by tsetse DNA. Selection of only engorged tsetse flies for the identification of host blood meals was to ensure the availability of host DNA in the blood meals since tsetse digestion of blood meals which usually occurs few days after ingestion may denature host DNA and impair its detection [16]. We also included only four species of vertebrate hosts in our assay because they were the commonest animals in the region where this work was conducted.

Tsetse feeding preference for cattle based on cattle size has been reported by Torr et al. [34]. Of all the vertebrate hosts included in our assay, cattle were the largest and this may explain why majority of the tsetse flies fed on them. The influence of host availability on tsetse feeding habits has also been documented [35]. This may also be a probable reason for the large proportion of pig blood identified by our study. Hunting dogs would have contributed to the number of tsetse flies that fed on dogs while activities like hunting, fishing, farming, and visit to rivers and streams would have exposed humans to the tsetse bites amounting to the number of tsetse that fed on humans.

Of particular concern are the dual and multiple feeding patterns involving more than one species of vertebrate hosts because this may be of great epidemiological importance in the area of spreading tsetse-borne infections between different vertebrate hosts including man in the region. Considering the proximity of this game reserve with the Fonten sleeping sickness focus of the Republic of Cameroon, there is the risk of trans-boundary tsetse flies acquiring* Trypanosoma brucei gambiense* and spreading the infection among humans and animal reservoirs in the region.

We concluded that* Glossina palpalis* is predominant and had higher infection rates.* Trypanosoma brucei* was more prevalent among tsetse flies. Tsetse flies fed predominantly on cattle and less frequently on humans and also showed mixed feeding habits. The risk of tsetse flies transmitting tsetse-borne infection between humans and other vertebrate hosts exists in the region.

## Figures and Tables

**Table 1:** Primer names and sequences for the amplification of mitochondrial cytochrome b in tsetse blood meals [16].

Host	Primer	Sequence 5′-3′	*T*_*m*_	Amplicon size
Human	Human741-F	GGCTTACTTCTCTTCATTCTCTCCT	66.08	334
Dog	Dog368F	GGAATTGTACTATTATTCGCAACCAT	62.30	680
Cattle	Cow121-F	CATCGGCACAAATTTAGTCG	58.35	561
Pig	Pig573-F	TTAGTCGCCTCGCAGCCGTA	64.48	453
Universal reverse	UNREV1025	GGTTGTCCTCCAATTCATGTTA	58.95	—

**Table 2:** Tsetse density and light microscopy infection rates in relation to study sites.

Study sites	Total catch	Average catch/trap	Average catch/day	Total dissected	Number infected	Infection rate (%)
Burto	142	0.99	5.94	117	8	6.84
Gindin Dutse	111	0.78	4.68	86	4	4.65
Goje	91	0.63	3.78	66	2	3.03
Serti	287	1.99	11.94	262	13	4.96
Total	**631**	**4.39**	**26.34**	**531**	**27**	**5.08**

**Table 3:** Tsetse infection rates in relation to *Trypanosoma* species identified.

Tsetse species	Total catch	Number dissected	Number infected	Number of pools	*T. brucei* (%)	*T. congolense* (%)	Mixed infections
*G. palpalis*	408 (64.7)	347 (65.3)	19 (5.5)	9	5 (55.6)	2 (22.2)	2 (22.2)
*G. tachinoides*	223 (35.3)	184 (34.7)	8 (4.3)	4	3 (75.0)	1 (25.0)	0 (0.0)
Total	**631 (100)**	**531 (100)**	**27 (5.1)**	**13**	**8 (61.5)**	**3 (23.1)**	**2 (15.4)**
*χ* ^2^	—	—	0.3168	—	0.4424	0.0120	1.0510
*p* value	—	—	0.5735	—	0.5060	0.9126	0.3054

**Table 4:** Distribution of *Trypanosoma* species in relation to study sites.

Study sites	Number of tsetse infected	Number of pools	*T. brucei*	*T. congolense*
Burto	8	4	3	1
Gindin Dutse	4	2	2	1
Goje	2	1	1	1
Serti	13	6	4	2
Total	**27**	**13**	**10**	**5**

**Table 5:** Distribution of mammalian blood in tsetse blood meals.

Study sites	Number of tsetse analyzed	Cattle (%)	Dog (%)	Man (%)	Pig (%)	Dual feeding (%)	Multiple feeding (%)
Burto	25	13 (52.0)	6 (24.0)	0 (0.0)	6 (24.0)	6 (24.0)	7 (28.0)
G/Dutse	25	12 (48.0)	4 (16.0)	2 (8.0)	7 (28.0)	2 (8.0)	2 (8.0)
Goje	25	6 (24.0)	5 (20.0)	3 (12.0)	11 (44.0)	4 (16.0)	3 (12.0)
Serti	25	14 (56.0)	2 (8.0)	1 (4.0)	8 (32.0)	12 (48.0)	5 (20.0)
Total	**100**	**45 (45.0)**	**17 (17.0)**	**6 (6.0)**	**32 (32.0)**	**24 (24.0)**	**17 (17.0)**
